# Performing publics of science in the COVID-19 pandemic: A qualitative study in Austria, Bolivia, Germany, Italy, Mexico, and Portugal

**DOI:** 10.1177/09636625231220219

**Published:** 2024-02-02

**Authors:** Helena Machado, Cláudia de Freitas, Amelia Fiske, Isabella Radhuber, Susana Silva, Christian O. Grimaldo-Rodríguez, Carlo Botrugno, Ralph Kinner, Luca Marelli

**Affiliations:** Department of Sociology, Institute for Social Sciences, University of Minho, Portugal; Laboratory for Integrative and Translational Research in Population Health, (ITR); EPIUnit - Institute of Public Health, University of Porto, Portugal; Institute of History and Ethics in Medicine, TUM School of Medicine, Technical University of Munich, Germany; Department of Political Science, University of Vienna, Austria; Department of Sociology, Institute for Social Sciences, University of Minho, Portugal; Centre for Research in Anthropology (CRIA-UMinho), Portugal; Psychology Education and Health Department, Western Institute of Technology and Higher Education (ITESO), Mexico; Research Unit on Everyday Bioethics and Ethics of Science, Department of Legal Sciences, University of Florence, Italy; European Institute of Oncology (IEO), Italy; Department of Development Studies, University of Vienna, Austria; Department of Medical Biotechnology and Translational Medicine, University of Milan, Italy; Life Sciences & Society Lab, Centre for Sociological Research, KU Leuven, Belgium

**Keywords:** COVID-19, mistrust, performativity, public trust in science, publics of science

## Abstract

Research about science and publics in the COVID-19 pandemic often focuses on public trust and on identifying and correcting public attitudes. Drawing on qualitative interviews with 209 residents in six countries—Austria, Bolivia, Germany, Italy, Mexico, and Portugal—this article uses the concept of performativity to explore how participants understand, and relate to science, in the COVID-19 context. By performativity, we mean the ways by which participants understand themselves as particular sorts of publics through identification with, and differentiation from, various other actors in matters that are perceived as controversies surrounding science: COVID-19 vaccination, media communication of science, and the interactions between governments and scientists. The criteria used to construct the similarities and differences among publics were heterogeneous and fluid, showing how epistemic beliefs about the nature of, and trust in, scientific knowledge are intermingled with social and cultural memberships embedded in specific contexts and across disparate places.

## 1. Introduction

Most studies assessing public attitudes toward science in the context of COVID-19 tend to see publics as in need of education in the understanding of basic processes of science in response to a global public health emergency (The Lancet Infectious Diseases Editorial Board, 2020; Wang et al., 2022). These studies are usually concerned with identifying and correcting knowledge and attitudinal deficits that might have an impact on risk perception related to COVID-19 (Krause et al., 2020; Savadori and Lauriola, 2022). As such, they tend to assume that attitudes toward science can be quantified and anticipated through associations with sociodemographic characteristics (Kaine et al., 2022), as well as with psychological and contextual elements (Bavel et al., 2020; Rutjens et al., 2021). Other studies link public trust to public dispositions to follow science-based recommendations (Bozeman, 2022; Devine et al., 2021).

A more pluralistic way of understanding how citizens relate to science in the context of COVID-19 demands recognition that not only are the terms “science” and “trust” polysemous and relational (Jennings et al., 2021; O’Doherty, 2022), but also that public positionings might be multiple and hybrid. Drawing on qualitative interviews with 209 residents in six countries—Austria, Bolivia, Germany, Italy, Mexico, and Portugal—we explore the complexity and dynamics of processes by which the participants understand, and relate to science, in the COVID-19 context. We take inspiration in social studies of science and technology (STS) that address the range and diversity of local, contextualized understandings of science and technology (Irwin and Michael, 2003; Martin and Donovan, 2015; Michael, 2009). We use the plural “publics” to refer to coalitions or hybrid groups characterized by heterogeneity and fluidity in their understandings of science. In particular, our article takes inspiration from the theoretical proposition of the STS scholar Mike Michael (2009) regarding the ways in which people use their own cultural and social resources to construct processes of identification and differentiation not only in relation to science but also in relation to other publics. In Michael’s (2009) words, “. . . this self-performative aspect of ‘doing being a member of the public’—the process by which laypeople enact themselves as publics through identification with, and differentiation from, various other actors—actors that include science but also, crucially, publics” ( p. 618). By use of cultural and social resources, we are referring to how values and cultural memberships are mobilized in the production of people’s views within a situated context, as well as to how they may be invoked for constructing and negotiating the production of truth claims about what is real and/or in whom to trust. The concept of enacting or performing (Epstein, 1996) publics has been used in studies aiming to understand the potential role of local or contextualized experiential knowledge in policy-making (see Goven and Morris, 2012; Nugroho et al., 2018), in case studies involving controversies about new science and technologies (Irwin and Michael, 2003; Michael and Brown, 2005), or about politicized issues such as biometrics (Amelung and Machado, 2019; Martin and Donovan, 2015). These studies show how laypeople relate to science by enacting claims through their own experiences and knowledge in order to constitute alternative stances in relation to institutionally sanctioned, expert scientific narratives. The performativity aspect of this dynamic and complex process relates to how, by understanding and relating to science, individuals are also making sense of themselves as belonging to a particular group of people who share views about science that is different from other publics.

By unpacking what is meant by publics and science in the context of the COVID-19 pandemic, we seek to explore how publics come into being and to illustrate the complexities of what publics are. We do so by looking into how the formation of publics articulates with forms of normative and relational engagement (Spahl et al., 2022) and with (mis)trust^1^ in relation to science-related issues perceived by the participants in this study as rife with controversy: COVID-19 vaccination, media communication of science, and government and science interactions. We argue that the criteria used by participants to enact themselves as publics in relation to science (Michael, 2009) in the COVID-19 context are heterogeneous and fluid, showing how epistemic beliefs about the nature of scientific knowledge intermingle with social and cultural memberships that are embedded in specific contexts and across disparate places.

## 2. Methods

This article results from the joint work of members of a qualitative, longitudinal and multinational study entitled Solidarity in Times of a Pandemic, or SolPan(+) as an acronym, that was carried out in Europe and in Latin America. It draws upon semi-structured interviews conducted in Europe with residents in Austria, Germany, Italy, and Portugal between October and December 2021, and in Latin America with residents in Bolivia and Mexico between August and December 2021. Most countries in this study are high-income countries (Austria, Germany, Italy and Portugal), one is an upper middle income country (Mexico) and another one is lower middle income (Bolivia) (World Bank, 2022). In selecting countries for this article, we strived to include countries with ranging levels of trust in science and scientists in the management of the COVID-19 pandemic (Wellcome Global Monitor, 2021). It was not our aim to engage in a comparison between national contexts where indicators are predetermined, but our analysis was sensitive to differences where they became apparent.

Participants were recruited using snowball and convenience sampling, in an effort to diversify sociodemographic characteristics (Table 1). We discontinued sampling when saturation concerning a broad range of views was reached. Although our sample is biased toward people with higher levels of education, the inclusion of data from six countries afforded greater sensitivity and theoretical saturation regarding the views of participants from particular socioeconomic and political contexts.

**Table 1.** Interviewees’ sociodemographic characteristics.

	Austria	Bolivia	Mexico	Germany	Italy	Portugal
Total of interviewees	55	27	25	40	24	38
Gender						
Female	36	17	18	21	19	19
Male	15	10	7	19	19	19
Age						
18–30	8	10	8	8	10	10
31–45	12	12	11	16	8	8
46–60	21	3	1	4	10	10
60+	14	2	5	12	10	10
Educational level						
<10 years	4	3	2	2	2	0
10–14 years	17	8	10	12	12	11
Higher education	34	15	12	26	10	27
No information	–	1	1	–	–	–

Invitations for participation in the study were made through the websites of the universities and research groups participating in the SolPan(+) consortium, as well as through email lists, social media, and personal contacts. Participants received in-depth information about the study and consent was obtained orally, directly before the interview. The consent and the interview were recorded. Only audio material was stored for transcription, and transcripts were pseudonymized and transcribed verbatim. Interviews were conducted online or in person.

Since the interview guide^2^ did not include questions directly addressing publics’ perception of science, the entire body of a total of 209 interviews (Austria = 55, Bolivia = 27, Germany = 40, Italy = 24, Mexico = 25, and Portugal = 38) was inductively analyzed to select relevant data in which the participants focused on science, scientists, and experts. Issues in relation to science that repeatedly emerged from the data concerned positions on COVID-19 vaccination, media communication, and government and science interactions. We followed a constructivist grounded theory approach (Bryant and Charmaz, 2012) that has been adapted for large-scale qualitative comparative research (Zimmermann et al., 2022). All interviews were coded using an inductively generated Master Coding Scheme developed by the SolPan(+) consortium data analysis group. This made data accessible for content-specific analytical work and helped the researchers familiarize themselves with the data. Interviews from each country were first analyzed separately and then combined and contrasted in team discussions. Quoted participants are anonymous, but each participant received a country code and a number provided after each direct quotation.

## 3. Performing publics of science in the COVID-19 context

Participants represented themselves as “affected publics” (Marres, 2007) by recognizing that they were implicated in a crisis—the COVID-19 pandemic—which prompted their engagement with matters related to science (Dewey, 1927; Wynne, 2005). In this context, the participants identified with or demarcated themselves from certain actors (other citizens, scientists, medical doctors, the media, policymakers) in specific controversies (Lippmann, 2002 [1927]). In discussing the formations of publics in this article, we draw on labels as heuristics^3^ to convey the positionings of specific publics in relation to vaccines, media communication of science, and government and science interactions, as the key issues around science that emerged in our data.

### COVID-19 vaccination: From supporting to questioning publics

When talking about science in the context of the COVID-19 pandemic, one of the most frequent topics mentioned by participants was the development of COVID-19 vaccines. Participants’ positionings ranged from adopting a posture of vaccination support that echoed a broader pro-science stance (Bauer et al., 2021), to engaging in critiques or specific inquiries regarding vaccination. Participants who took science at face value on this matter frequently referred to unvaccinated people as vaccine skeptics who would often not trust scientific processes and findings in a broader sense (Barker et al., 2021: 9). Other participants demarcated themselves from this positioning, either by being inquisitive and seeking out contrasting scientific information before making decisions about vaccination, or by questioning medical and scientific approaches to the COVID-19 pandemic. There were also participants, both among the “supporting” and the “questioning” publics, who engaged with complementary medical knowledge (often outside dominant understandings of Western science), conveying nuanced views and fluid positionings that eschew binary categorizations.

Those participants who highlighted the merits of the COVID-19 vaccines felt that science could be trusted, and that it should be recognized and celebrated as an available solution to the predicaments of the pandemic. Pronouncements about trust and faith in science showed how these participants engaged in a lay public defense of science in which COVID-19 vaccination was perceived as an extraordinary expression of science’s capabilities (Adhikari et al., 2022):

I have a fundamental belief in science. And that’s why I didn’t have a problem getting vaccinated. (AT28)

I really rely on science and scientists and their opinion on it [. . .] how dangerous the disease is and how well the vaccination protects us [. . .] I was satisfied with it, because scientists had their say. (DE08)

It was an extraordinary advancement [development of Covid-19 vaccines], and welcome to science! [Covid-19 vaccines] are for the good of humanity and we must also embrace it as an outstanding achievement. (PT15)

In our study, some participants who considered themselves supporters of COVID-19 vaccination, talked about others who refused to be vaccinated as holding antiscientific beliefs and hostility toward science (Barker et al., 2021). The plurality of publics engaged in vaccination refusal tended to be perceived by some participants as uneducated, ignorant, and annoying, as opposed to vaccinated people whom they described as well-informed:

People’s stupidity bothers me . . . This hostility to science in highly praised Europe and in Austria bothers me above all [. . .] I mean, what went wrong in the education system? (AT55)

Astra Zeneca vaccinated, cross-vaccinated [. . .] These are all well informed people, who have great trust in how science works. If they [scientists] say so, I can’t judge that better, I trust them [. . .] in that respect there was very large approval in my bubble [. . .] [There are] unvaccinated people coming up with such completely new positions that I’m now realizing how this clearly irritates me. (DE22)

At the beginning many people doubted . . . in fact, there’re people who said: “It’s a government thing.” But [after] so many deaths, so many losses, you said: “How is it possible? Of course it exists”. But, there were people who, I don’t know if out of ignorance [. . .] said: “No. Those are things of the government,” that is, the virus does not exist. (MX23)

For many participants, support for vaccination was equated with moral correctness, that is, with publics doing the right thing to protect the common good and to return to “normality” (Fiske et al., 2022; Paul et al., 2022). At the same time, participants also likened this positioning to adopting a stance of believing in and supporting science:

I feel more of that group thing, if we all take [COVID-19 vaccines] and why not believe in it? Again, why not believe in science and vaccines when we do so many other things that are much worse, right? I smoke, I drink. (PT26)

[When] vaccines came out, there was resistance by many people. [. . .] There were also people who were vaccinated [. . .] I speak personally, I received the two doses of the vaccine and I’m aware that if we want to return to something normal we should opt for the vaccine. (BO02)

There were also interesting overlaps among people who believed in science, supported vaccination and, simultaneously, engaged with other sources of complementary medical knowledge. Indigenous knowledge was mentioned by some participants as credible knowledge and expertise to deal with COVID-19. The following quotations illustrate the positive value attributed by some respondents in Bolivia to traditional medicine being practiced both by the Indigenous population and by healthcare practitioners in medical centers (Mathez-Stiefel et al., 2012):

I would say that for us [the COVID-19 pandemic] was a lesson for not forgetting our medicinal plants, ancestral plants. (BO11)

So, if someone needs something, they go to the medical center and, well, the medical center of our community is allied to traditional [Indigenous] medicine. The medical doctors and nurses are the ones who know what [medicines] to take in each case [antibiotic or medicinal herb] [. . .]. (BO05)

Other participants demarcated themselves from the vaccine-supporting publics by referring the need to be cautious, inquire further and adopt a watchful waiting attitude. These questioning publics frequently manifested a positioning of lay expert people who sought further, diverse scientific information before deciding about vaccination. In this positioning, some participants refused the label of denialists (Zimmermann et al., 2022) that other people, the media, or governments attributed to the unvaccinated:

[. . .] the discrimination of the unvaccinated, who are still waiting due to a thought process, and we’re currently being lumped together with brainless [right-wing populist party] voters. (AT07)

Something very serious happened, which was to create a great duality of narratives: calling anyone who questions anything a denialist and this soon being on the cover of the newspaper [. . .]. I do not say that it [the virus] does not exist, I do not say that vaccines are not effective at all, but I question it, right? (PT22)

Some of the participants who engaged in a questioning positioning invoked the value of alternative knowledges and non-biomedical approaches and criticized the media’s exclusive focus on vaccination and its neglect of preventive measures and alternative (therapeutic) options to COVID-19 vaccines. They also pointed out how the media disqualified alternative approaches to deal with COVID-19 by either condemning people who use traditional medicine for forsaking medical doctors’ advice or by categorizing people with different views as denialists:

I feel I have a broader vision of health than the one the media bombards [us with]. [. . .] Sometimes, it also seems that it’s like the cliché of: “Oh, the ladies who don’t pay attention to the doctor and take this little herb [traditional medicine] and that will get rid of it.” [. . .] I think there’re intelligent ways of knowing how to do it, not that these ladies are not, but rather the way people speak about them seems that they’re not. (MX15)

I question the ways in which we can control [COVID-19]. There may be several, but at the moment, only one is imposed on us, which is vaccination and this has created a great duality at the social level. [. . .] I think that the media itself only adopts this type of narrative, leaving no room for other issues, without these issues being labeled denialism. (PT22)

For some participants, healthcare professionals were perceived as having additional responsibility in protecting the credibility of science (Gieryn, 1983). They spoke of being concerned about health professionals who refused to be vaccinated or who raised doubts about COVID-19 vaccination. Hence, vaccination refusal by some medical doctors was denounced as a signal of “normative chaos” (Barker et al., 2021: 17), in the sense that these professionals might have a negative influence on the population by spreading confusion and increasing indecision. One interviewee criticized the medical doctors involved in “the denialist movement” claiming that insufficient knowledge makes people vulnerable to the transmission of anti-vaccine information and its negative effects:

The denialist movement . . . I think this is profoundly stupid. I don’t believe there’re doctors who have a certain scientific training and who are against it [COVID-19 vaccines]. [. . .] Most of them think it’s good to take the COVID-19 vaccines and half a dozen say that it’s not good, that there will be problems [. . .] They will influence certain people who are more vulnerable, who do not have knowledge [. . .] They shouldn’t. (PT01)

Another interviewee referred to a friend who, despite being a scientist, opted for taking “home remedies” rather than getting vaccinated. This behavior was considered both irresponsible and paradoxical given its incoherence and contradiction with scientific knowledge:

I have a friend who is a chemist at the university. She’s a chemist, super smart [. . .] she says she didn’t get vaccinated and you say: “How can you not get vaccinated if it can save your life?” [. . .] She, says: “I drink an aloe vera shake in the morning” [. . .] lots of home remedies. [. . .] with that she feels protected [. . .]. I mean, where did that woman’s intelligence go? [. . .] I consider it irresponsible. (MX23)

Similarly, the following participant equated vaccination with what was both the epistemic (true) and moral (righteous) answer to the pandemic by arguing that the emergency approval of vaccines should have not been questioned by healthcare professionals:

[. . .] many people from the health sector who say [. . .] that we can’t know what’s good and what’s not good. And this group is currently completely excluded and I’m very sorry, but it is understandable, because this experiment of emergency approval of vector and mRNA vaccine must not be called into question. (AT07)

Another participant reinforced her pro-science positioning by rebutting what she considered to be the unfounded characterization of COVID-19 vaccines as a rushed product, suggesting instead that vaccines were problem-solving technologies that built on a long history of science-making (Harrison et al., 2022):

There was an absurd notion of having produced the vaccines with what is said to have been great speed. This simply is not true. It was perhaps a little faster than usual, but a little, nothing special. (PT15)

As other work has demonstrated, issues like vaccination defy binary framings (Zimmermann et al., 2022). Similarly, the positions described here concerning vaccination were not fixed, but rather malleable and relational (Fiske et al., 2022), amid the fluid territory of public relationships with science. The criteria used by the participants to construct similarities and differences among publics in regards to COVID-19 vaccination were heterogenous and fluid, showing how epistemic beliefs about the nature of scientific knowledge intermingled with social and cultural memberships embedded in situated contexts. Moreover, our analysis reveals how participants enacted themselves as different publics also in relation to divergent views of science as either an autonomous space clearly demarcated from the “others” of society, politics, and the economy, or an enterprise fully embedded in society and therefore imbued with the normative preferences and interests of the actors involved in it.

### Media communication of science: From confused to proactive publics

Science in the COVID-19 pandemic unfolded in real time under intense public scrutiny, raising particularly pressing doubts about the credibility of scientific establishments (Dunwoody, 2020; Prasad, 2022). Challenges to the cultural authority of science and the making of boundaries between science and non-science (Gieryn, 1983) were especially prominent due to the intense media attention devoted to scientists and experts (Mach et al., 2021). Several participants noted how the uncertainty surrounding the COVID-19 pandemic generated an overflow of often inconsistent information that affected different publics in dynamic and heterogeneous ways: some people were “confused,” while others acted proactively to navigate the maze of information.

Confused publics were described by the participants as people who were overwhelmed by the quantity and contradictory nature of information shared by the media. According to the following participants—who self-described as being part of confused publics—one way to deal with the overflow of conflicting information was by giving up watching the news or listening to scientists:

[On the television] There was a doctor who said: “No, you have to do this.” And another said: “Yes, you have to do that.” [. . .] It was so difficult to know what was true and what wasn’t. That has confused [people] [. . .]. I tell you, I’m not the only one, many people have stopped watching the news. (BO02)

Scientists still don’t speak in a single voice. I don’t want to hear them, I refuse. Otherwise, my head blows! (PT14)

When faced with overwhelming information, some participants who considered themselves to be part of the proactive publics, adopted a lay expert role. They took scientific matters into their own hands and looked up scientific information online or in the news which they perceived to be trustworthy in order to reject non-credible media. They also noted how people with limited resources were less equipped to undertake a critical stance and to assess the reliability of the information conveyed by the media about science:

I’m trying to find the original sources [about COVID-19]. I prefer to read the original sources. I don’t believe the press people. (DE23)

I had an opportunity to develop a critical attitude, for personal reasons, for educational reasons . . . but I am aware that, for instance, the elderly who had less opportunities from the educational viewpoint . . . maybe they can get impressed [by media], without having the opportunity to object when someone [an expert] says: “This is better than that.” (IT15)

For proactive publics, the lack of accurate and intelligible information in the media regarding the SARS-CoV-2 virus and COVID-19 vaccines caused at least two interrelated negative impacts. First, conflicting information about an unknown virus pushed people to accept what were often unpleasant changes to their lives out of resignation—people apparently compensated for uncertainty and ambiguity by showing deference to scientific authority (Brossard and Nisbet, 2006). Second, the communication of science in the media was expected to occur in alignment with the principles of neutrality and quality resonating with scientific knowledge (Takahashi and Tandoc, 2016). However, participants claimed that media outlets failed to inform the public accurately about the potential side effects of COVID-19 vaccines causing many people to refrain from vaccination:

Suddenly you had to know what type of virus it was, what type of contagion, care [. . .] So many people resigned to making changes in their lives for prevention. I think those were the failures, not having adequate communication of what was happening and in a digestible way. Because we listened to doctors, we listened to specialists, and many people didn’t digest what they were saying. (MX22)

So, so much was ruined at the beginning, which is still noticeable today, because the vaccination rate is still so low. But a lot could have been saved simply by providing comprehensible, neutral, quality-assured information. Someone should have explained to people that there are also sinus thromboses if I have not been vaccinated. (AT28)

Other participants defended the need for a more authoritative science (Post et al., 2021), in which professionals would provide univocal opinions and convey more certainty (Barker et al., 2021; Gauchat, 2011). In this view, the opening up of the uncertainties of science and areas of still-undone science (Hess, 2016; McGoey, 2012) was perceived as affecting the credibility of the scientific community as a whole. At the same time, limited consonance in the scientific knowledge shared by the media was seen as an obstacle to communicating science properly to laypeople who were perceived as confused publics:

I think that many people were confused because they listened to specialists who said diametrically opposite things and questioned: “Is this science?!” [. . .] I didn’t always see science at its best in terms of communication. (PT18)

Perhaps [. . .] less messy communication like we’ve seen, especially on television [. . .] it would have helped to pass on the importance of vaccination. (IT14)

Unlike those who argued the need for an authoritative science, other participants expressed the desirability of refusing one-sided media narratives and engaged instead in a positioning of accepting the epistemic uncertainty of science:

[People tend to] think that only one narrative is correct. I think that life doesn’t work that way, medicine doesn’t work that way, [and] science doesn’t work that way. (PT22)

Our results show that participants’ views on the communication of science about COVID-19 emerged from their understanding of the type of relationships established between scientists and the media, and scientists and policymakers (Post et al., 2021), along with expectations about the nature of scientific knowledge. One sharp distinction between confused publics and proactive publics derived from how participants articulated their own informational needs with their views on the nature of science: people seeking more definite information embraced an understanding of scientific knowledge as stable and certain, and manifested discomfort with disagreement among scientists. They also claimed the need for clear communication, which was seen as equivalent to the information they needed to navigate the COVID-19 crisis. In contrast, the publics’ adopting inquisitive stances and lay expert positionings emphasized the inescapable uncertainties of scientific knowledge and showed willingness to form opinions based on their own judgments of what was credible and non-credible scientific information.

### Government and science interactions: From trusting to mistrusting publics

The COVID-19 pandemic required scientists to work alongside policymakers more closely than in any other recent event. While in normal circumstances, scientific experts provide evidence and inform political decisions largely outside of public perception and debate (Collingridge and Reeve, 1986; Wynne, 2007), in the extraordinary circumstances of the COVID-19 pandemic, this function of expert advice turned out to be highly contentious (Weingart et al., 2022). At the same time, the ways in which different countries mobilized scientific expertise to manage the pandemic differed considerably. Cross-national studies on policy-making and public trust in science in the COVID-19 context (Algan et al., 2021) showed that trust in official scientific advice often correlates with trust in government. However, we found a more complex picture in this study: instead of a straightforward association between (mis)trust in government and (mis)trust in official expert advice, our respondents engaged in credibility judgments by demarcating science from non-science (Gieryn, 1983).

We found several dynamic instances of mistrusting publics among participants in all countries, where the interviewees pointed to the vested political leanings of researchers, the urge of scientists to be in the media, or economic interests of major actors in the field (e.g. pharmaceutical companies) as factors that trigger mistrust in science (Pinto, 2020):

. . . the State forces me to get vaccinated because if I don’t do that, I am not allowed to do anything. [. . .] There is something on which they have come to an agreement, in the sense that money always makes the difference. Pharmaceutical companies for sure have influenced these decisions. It’s sad, but we live in a world of corruption, and everybody can see very clearly what’s happening. (IT03)

Scientists who wore the scientist hat most of the time, what I heard them say was unscientific [. . .] [they attempted] to use that stage [media] to obtain more funding, to pressure public authorities to finance them [. . .] they were hired to justify government policies. [. . .] how did science go beyond its sphere of competence to [engage in] political intervention? (PT16)

It’s always the same virologists who are commissioned by the government to talk, but you don’t know what you can or should believe in the meantime. [. . .] I find [a collective of specialists] more credible than the isolated personalities who are more or less bought somewhere. (AT43)

In contrast, the participants based in Germany frequently assumed a stance of trusting publics, which is in line with results from past studies (Hangel et al., 2022; Wellcome Global Monitor, 2021), referring to their trust in key expert figures providing scientific support to government measures (Leidecker-Sandmann et al., 2022):

In a situation like this, I think it’s very important that the scientists kind of decide, well, not necessarily where to go, but how the facts are, and that politicians are guided by scientific opinions and do what the scientists recommend. (DE15)

However, trust was relational (O’Doherty, 2022) in the sense that the participants referred to their own experiential knowledge (Paul et al., 2022; Spahl et al., 2022) to assess the credibility of scientists’ advice, and, simultaneously, tended to trust experts who had a good reputation and with whom they were familiar:

I spoke to people I knew, my colleague who dealt with it [vaccination] quite intensively. Then she told me: “Drosten has a podcast, listen to it, it’s really good.” And yes, I have checked that I get at least selectively active information from experts that I also trust or whose education or field of research I also somehow trust. (DE03)

. . . the press, this briefing every week with the chairman of the Robert Koch Society [. . .] I really had faith in the government action and the media also contributed to this. (DE32)

The strong presence of positionings of trusting publics among respondents in Germany did not exclude critical views, with participants adopting a stance of lay expert publics by stressing the importance of making their own risk assessment, which was often associated with the perception that further scientific evidence was needed, for example, on the effectiveness of vaccination (Zimmermann et al., 2022):

I hear Mr. Drosten and Mr. Lauterbach say you have to be vaccinated twice and then it would be best to get infected with Corona again, so that you have the best possible protection against Corona. Please, what is that, some stupid logic? So, I can’t take seriously what is publicly claimed there. (DE30)

The case of Mexico emerged as a singular example of how sharp inconsistencies in governmental action inspired the constitution of both mistrusting and trusting publics of science, depending on whether those publics’ positionings were preferentially guided by their political affiliations or by the idea that policy should be informed by interest-free science:

[The authorities] were giving mixed messages. On the one hand, the Undersecretary of Health was saying that we had to be careful and, on the other, the President was giving the opposite message that nothing is happening. And, unfortunately, this person has a lot of influence over the people, [. . .] and, unfortunately, many pay more attention to the President than to the doctor. (MX03)

In this quotation, the interviewee highlights the contradiction between the positions of political-electoral and scientific figures, noting how it divided the Mexican population between those who believe in science and those who believe in the president.^4^ The Mexican media cast doubt upon the political affiliations of scientific experts entrusted by the Federal Government with communicating public health measures, reporting new cases and sharing relevant scientific findings about COVID-19. This dubious relationship between political actors and “official” scientists not only influenced the way in which the publics were configured according to their political partisanship and adherence to scientific findings, but it also affected their perception of risk of contagion, as some publics conceived the COVID-19 as a government invention instead of a scientifically describable biological hazard (Chayinska et al., 2022).

Participants’ views about the role of science, and scientists, in their relationship with governments showed positionings that privileged scientific knowledge and expertise as essential to policy-making, leading to dynamic and malleable positions as trusting and mistrusting publics. For many participants, science should not stand above scrutiny, while, at the same time, they tended to place trust when they perceived scientists to be clearly disinterested from and uninfluenced by political, economic, or social interests (Mihelj et al., 2022).

## 4. Conclusion

This qualitative study showed that when individuals understand, and relate to, science, they might also be understanding, or relating to, themselves as particular sorts of publics (Michael, 2009: 619)—they perform publics. The performative nature of constituting publics was evidenced by addressing how the participants made sense of themselves as belonging to a particular public (a group of people who share views about the role of science) that was different from other publics. A plurality of dynamic publics emerged in relation to three matters—COVID-19 vaccination, media communication of science, and government and science interactions. These self-performing publics of science range from supporting to questioning publics, from confused to proactive publics, and from trusting to mistrusting publics.

The criteria used to construct the similarities and differences between these publics were heterogeneous and fluid. One strategy enacted by some participants was self-identification with a stance of vaccination defense, and references to unvaccinated people as vaccine skeptics who do not understand what science is and/or are hostile toward science. Vaccine supporting publics expressed faith in science and felt they could trust scientists, partly because of their extraordinary achievements (i.e. the development of a vaccine in record time) and partly out of moral correctness and the need to get back to “normal.” Other participants demarcated themselves from this stance by adopting a questioning positioning and a posture of lay expert publics, being inquisitive and proactively seeking and contrasting scientific information before making decisions about vaccination. And yet others, both among the supporting and questioning publics, claimed the value of complementary medical knowledges to deal with the COVID-19 pandemic, adopting fluid positionings that reject binary categorizations.

In the face of radical uncertainty, and often contradictory information about COVID-19, many participants identified themselves, or other people, as either being confused by the overwhelming flow of information, or being proactive in navigating the maze of information and guidance provided in connection to the pandemic. While some participants took scientific advice and governmental COVID-19 recommendations at face value, others proactively tried to discern what comprised credible scientific information and who were credible experts. In these practices of demarcation of science from non-science, some participants represented themselves as lay expert publics (i.e. taking scientific matters into their own hands and making credibility judgments). Altogether, the performance of particular sorts of publics showed how the intense media attention devoted to science and experts in the COVID-19 context raised particularly pressing challenges to the cultural authority of science. It also uncovered divergent views about the nature of scientific knowledge, which participants perceived to be inscribed within a continuum ranging from outright certainty and stability to inevitable uncertainty and change.

Finally, the extraordinary circumstances of the COVID-pandemic that led to the high public visibility of scientific experts informing political and governmental decisions, turned out to be highly contentious. The ways in which governments in the six countries included in our study mobilized scientific expertise differed considerably. However, we found a complex picture that goes beyond the straightforward correlation between (mis)trust in government and (mis)trust in official expert advice, which is frequently assumed in surveys about public attitudes toward science. Our respondents engaged in credibility judgments that enacted a plurality of trusting and mistrusting publics. Trust in science was frequently associated with highly respected and familiar scientists providing official advice free from secondary political, reputational, or financial interests. In contexts in which the participants perceived the actions of governments as being based on scientific advice of this kind, science seemed to function as a form of guidance for the trusting publics. Conversely, views about factors such as the vested political leanings of researchers, the assumed urge of scientists for media attention, or potential economic interests of major actors in the field (e.g. pharmaceutical companies) triggered mistrusting publics.

Participants’ views were related to values that made sense in the specific contexts of their everyday lives, and emerged in different ways across the countries in this study. It was not our aim to conduct a cross-country comparative study, and therefore nationality did not play a role in the analytical approach nor in the discussion of results. Instead, this article shows how the diverse range of stances were remarkably similar across the six countries, which is why we only refer to specific country cases whenever they hosted particularities that stood out.

The relative, situated forms of personal and collective identification and demarcation—the performativity of publics—showed how epistemic beliefs about the nature of scientific knowledge are intermingled with social and cultural memberships. These heterogeneous elements generated new differentiations and divides related to the special circumstances of the COVID-19 pandemic, performing publics who do not host a fixed composition but, rather, are situated along a set of continuums (supporting to questioning, confused to proactive, trusting to mistrusting). Adopting a performativity approach has thus enabled us to move beyond approaches that address trust in science and scientific literacy as individual dispositions that can be measured and, instead, conceive of these views as a relational process situated within cultural and epistemological frameworks that relate to several aspects, including the participants’ beliefs about the nature of scientific knowledge (Howell et al., 2020), and to relations of trust or mistrust between lay individuals and toward key social actors, namely scientists, the media, and governments.

## Notes

1. We use the concept of mistrust (instead of distrust) following Jennings et al. (2021) who discussed how the concepts of trust, distrust, and mistrust could be applied for assessing citizens’ perceptions of COVID-19-related threats, and their responses to it. According to the authors, mistrust is not based on a settled belief. Instead, it involves a continuous process of feedback and updating against trust and trustworthiness.

2. The interview guide included several questions related to participants’ views and lived experiences of the COVID-19 pandemic. The questions that raised interviewees’ views about science were related to COVID-19 vaccination, media coverage of COVID-19, and governmental measures to contain the COVID-19 pandemic.

3. We use the term heuristic as a conceptual short-cut to summarize the range of characteristics and positionings by which publics identify or demarcate themselves from science and/or other publics.

4. Mexican citizens were often confronted with conflicting guidance from authorities and highly disparate policies across state lines. For example, President Andrés Manuel López Obrador created some distance between himself and actions announced by members of government to contain COVID-19. Nevertheless, at times, he spoke in support of such measures, while, at other times, he advocated for citizens to lead life as normal. He also suggested that he did not need to worry about the virus because he was protected by religious amulets (Dunn and Laterzo, 2021).
